# When Is Imaging Appropriate for a Patient With Low Back Pain?

**DOI:** 10.31486/toj.20.0077

**Published:** 2020

**Authors:** Brian Lancaster, Joshua Goldman, Yuka Kobayashi, Andrew W. Gottschalk

**Affiliations:** ^1^ Department of Family Medicine, Division of Sports Medicine, University of California, Los Angeles, Los Angeles, CA; ^2^ Department of Family Medicine, Oregon Health and Science University, Portland, OR; ^3^ Department of Orthopedics, Sports Medicine Institute, Ochsner Clinic Foundation, New Orleans, LA

## CASE

A 48-year-old pickleball player comes to the primary care clinic complaining of low back pain for 5 days. He was playing in a game when he reached for the ball and noted immediate sharp pain in his right lumbar region. He has been managing the pain with relative rest, alternating ice and heat, and appropriately dosed ibuprofen. However, he is concerned that the sharp pain has not resolved. He asks the provider to “get pictures” to make sure “everything is okay.”

## BACKGROUND

Low back pain is the second most common complaint in the United States, with a lifetime incidence of 80% to 85%.^1^ Internationally, the prevalence of back pain has been reported as high as 20% in first-world countries.^1^ Acute low back pain, defined as pain of less than 6 weeks’ duration, is a self-limited condition that does not require imaging for uncomplicated cases.^2^ Uncomplicated cases are typically defined as a lack of red flag signs, symptoms, and concerns (Tables 1 and 2), with resolution of presenting symptoms within 6 weeks of conservative medical management.^1^ However, inconsistency and confusion remain with regard to appropriate utilization of imaging modalities when patients present with new-onset back pain. Inappropriate imaging leads to increased healthcare costs, risk to patients through unnecessary radiation exposure, and potentially unnecessary care.^3^

**Table 1.** Red Flags for Malignancy

Signs, Symptoms, and Concerns
History of malignancy
Strong clinical suspicion
Unexplained/unintentional weight loss
Age >50 years
Failure to improve with 4-6 weeks of treatment
Pain at rest
Malaise
Fever
Reduced appetite
Rapid fatigue
Progressive symptoms
Multiple cancer risk factors
Paraparesis

Note: Adapted from Verhagen et al.^6^

**Table 2.** Comprehensive Red Flags and Reasons for Concern as Defined by the American College of Radiology^1^

Red Flag Symptom	Concern
History of malignancy	Malignancy
Unexplained weight loss	Malignancy
Immunosuppression	Infection, malignancy
Urinary infection	Infection, malignancy
Intravenous drug use	Infection, malignancy
Pain not improved with conservative care	Infection, malignancy
Prolonged use of steroids	Fracture
History of significant trauma	Fracture
Minor fall/heavy lift in osteoporotic/elderly individual	Fracture
Acute onset urinary retention or overflow incontinence	Cauda equina syndrome, severe neurologic compromise
Loss of anal sphincter tone or fecal incontinence	Cauda equina syndrome, severe neurologic compromise
Saddle anesthesia	Cauda equina syndrome, severe neurologic compromise
Global or progressive motor weakness in lower limbs	Cauda equina syndrome, severe neurologic compromise

## REVIEW OF EVIDENCE

Physicians order imaging on nearly one-third of patients not meeting appropriateness criteria set forth by the American College of Radiology (ACR).^1^ A systematic review and metaanalysis of 31 studies that included 1.2 million patients evaluated physician imaging practices between 1995 and 2017.^4^ The authors found that 34% of lumbar imaging was inappropriate based on the absence of symptoms for serious pathology such as fractures, severe neurologic compromise, and malignancy. Conversely, patients who presented with red flag signs/symptoms or physician-reported concern for serious pathology were not appropriately imaged in 65.6% and 60.8% of cases, respectively.^4^

A comprehensive evaluation looking for red flag signs and symptoms remains a cornerstone of the initial assessment of acute low back pain. As such, the American College of Physicians and the American Pain Society endorse screening for red flag signs/symptoms and a trial of management without imaging in adults without risk factors.^5^ However, the true utility of these signs and symptoms is not readily understood in the primary care setting. A 2017 systematic review of 33 publications that included 13 different red flags endorsed by 16 guidelines found that a history of malignancy and high clinical suspicion were the only 2 red flags of 7 with acceptable diagnostic accuracy for malignancy.^6^ Of the 33 publications, the studies that evaluated the 7 signs and symptoms with the most acceptable diagnostic accuracy showed moderate quality evidence. The positive likelihood ratios for diagnostic accuracy of malignancy were reported to range from 6.4 to 15.3 for history of malignancy and 12 to 54.2 for high clinical suspicion. However, high clinical suspicion is a subjective measure, and the cues the physicians used to predict malignancies were not reported in the studies.^6^

An important note is that the prevalence of serious pathology in the primary care setting is low, and prospective cohort studies estimate the prevalence of spinal malignancy to range between 0% and 0.7% in patients presenting with low back pain.^6^ Radiographic studies of patients referred by primary care providers for low back imaging corroborate the low prevalence of spinal malignancy, with imaging-confirmed prevalence ranging from 0.2% to 0.7%.^6^ The low prevalence of serious pathology causing low back pain is insufficient to warrant imaging on all patients.

The ACR accordingly recommends imaging for low back pain in (1) patients who have had up to 6 weeks of medical management and physical therapy for low back pain without improvement in symptoms, and (2) patients with red flag symptoms for serious pathology.^1^

For patients meeting one of the above criteria for imaging, the choice of imaging should be guided by the suspected pathology (Table 2). Magnetic resonance imaging (MRI) of the lumbar spine is the modality of choice for the majority of patients requiring imaging, especially if the suspected pathology is malignancy or neurologic compromise, per the ACR appropriateness criteria. The use of intravenous contrast with MRI is generally only appropriate for patients with a history of prior lumbar surgery or cancer patients with concern for epidural or intraspinal disease.^1^ Lumbar spine radiographs are of limited utility and are generally only indicated when fracture is a concern because of a history of trauma or prolonged steroid use.^1^ Computed tomography should be reserved for patients with a contraindication to MRI.

## CASE RESOLUTION

After taking the patient's history and performing an examination, the provider determined that the patient had no red flags. The physician explained to the patient that imaging was unnecessary and provided the evidence presented above. The patient verbalized understanding of the reasoning and agreed to start a home exercise program. Four weeks later, the patient sent a message stating that his pain had improved. The decision to forego imaging saved the patient healthcare costs and radiation exposure.

## Notes

This article meets the Accreditation Council for Graduate Medical Education and the American Board of Medical Specialties Maintenance of Certification competencies for Patient Care and Medical Knowledge.
